# Development of Polarization-Insensitive THz-to-IR Converters for Low-IR-Signature Target Detection and Imaging

**DOI:** 10.3390/s24175614

**Published:** 2024-08-29

**Authors:** Berat Aytaç, Asaf Behzat Şahin, Hakan Altan

**Affiliations:** 1 Roketsan Missiles Inc., 06780 Ankara, Türkiye; 2 Department of Physics, Middle East Technical University, 06800 Ankara, Türkiye; haltan@metu.edu.tr; 3 Department of Electrical and Electronics Engineering, Yıldırım Beyazıt University, 06010 Ankara, Türkiye; absahin@aybu.edu.tr

**Keywords:** THz-to-IR converter, THz, metasurface, IR camera, temperature difference, low-IR-signature target, incoherent source

## Abstract

A THz-to-IR converter can be an effective solution for the detection of low-IR-signature targets by combining the advantages of mature IR detection mechanisms with high atmospheric transmittance in the THz region. A metallic metasurface (MS)-based absorber with linear polarization dependence based on a split-ring resonator (SRR) unit cell has been previously studied as a preliminary example of a THz-to-IR converter structure in the literature. In this simulation-based study, a new cross-shaped unit cell-based metallic MS absorber structure sensitive to dual polarization is designed to eliminate linear polarization dependency, thereby allowing for incoherent detection of THz radiation. A model is developed to calculate the temperature difference and the response time for this new cross-shaped absorber structure, and its performance is compared to the SRR structure for both coherent and incoherent illumination. This model allows for understanding the efficiency of these structures by considering all loss mechanisms which previously had not been considered. It is found that both structures show similar performance under linearly polarized coherent illumination. However, under incoherent illumination, the IR emittance efficiency as gauged by the temperature difference for the cross-shaped structure is found to be twice as high as compared to the SRR structure. The results also imply that calculated temperature differences for both structures under coherent and incoherent illumination are well above the limit of the minimum resolvable temperature difference of the state-of-the-art IR cameras. Therefore, dual-polarized or multi-polarization-sensitive MS absorber structures can be crucial for developing cost-effective THz-to-IR converters and be implemented in THz imaging solutions.

## 1. Introduction

**Figure 1:**
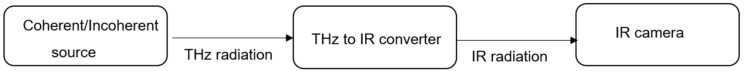
The working principle for detection of coherent/incoherent THz source with IR camera by using a THz-to-IR converter.

The detection of targets with low-infrared (IR) signatures is a topic of interest, and due to scenarios where atmospheric conditions can adversely affect IR transmission, new detection methods based on longer wavelength regions of the electromagnetic spectrum are needed. Targets with low-IR signatures can be detected under adverse conditions with appropriate detection mechanisms in TeraHertz (THz) bands (300–10,000 GHz) [1,2,3,4,5,6,7]. Typical THz detection methods have some drawbacks because of their complexity, high fabrication cost, and possible cooling requirement when compared to modern IR detection techniques. On the other hand, IR detection technologies are extremely sensitive and have considerably matured. The advantages for both IR and THz can be combined with the usage of a THz-to-IR converter, as an intermediary component, allowing for a cost-effective solution for the detection of low-IR-signature targets using IR sensing technologies. The general principle of THz-to-IR conversion is an intermediary device placed between the source and IR detector that absorbs the THz radiation well and conducts and radiates the heat with high efficiency, as shown in Figure 1 [1]. At the same time the speed in detection and conversion is also a relevant parameter which needs to be optimized. These constraints limit the types of THz absorbers that can be used. It turns out that 2D metamaterial structures, namely metasurfaces, perform well in terms of high absorption and a low profile allowing for thin, low-mass converter structures to be fabricated, thereby improving the detection speed [8].

In previous studies, Kuznetsov et al. [1,2] demonstrated a multilayered THz-to-IR converter structure designed for an operating frequency at 300 GHz. The absorber metasurface structure is based on a split-ring resonator (SRR) unit cell array and is composed of a metallic metasurface (MS) thin-film absorber layer, a dielectric substrate, a metallized ground layer to maximize absorption, and a dark emissive layer based on graphite. The resonant absorption of the THz wave from the converter causes heating and IR emission, which can be measured by an IR bolometer camera. Their demonstration showed that such devices can be successfully implemented for THz conversion studies; however, a full theoretical and numerical treatment was not performed on the device parameters, leaving room for optimization. In particular, the MS structure was sensitive to a single linear polarization state and thus the implementation of such structures is ineffective for multi- or unpolarized THz sources. Metallic planar metamaterial THz-to-IR converter MEMS-based structures with full treatment of the heat conduction and loss mechanisms based on the heat balance equation were performed to calculate temperature difference (ΔT) in the study by Alves et al.; however, their structure was optimized for the high-frequency THz region where the metallic planar metamaterial structure exhibits near-perfect absorption [9]. This latter study used the heat conduction and loss analysis by Padilla et al. [3], which is not based on a metallic MS absorber layer. When all of these studies are compared, it is found that a full treatment of a metallic MS absorber-based THz-to-IR converter that particularly works in the low-frequency end of the THz spectrum where atmospheric propagation effects are less severe has not been performed [5]. MS is not the only method that can be used for THz-to-IR conversion; metal nanoparticles also have been shown to be useful for measurement of THz sources [10]. Furthermore, one important point that stands out is that none of these previous studies study the conversion process for incoherent THz sources, which is important for passive detection scenarios where background illumination or THz emissivity of the target can be utilized. Our previous study [4] showed that to detect THz radiation from an incoherent passive source, the detector should be polarization-insensitive. As a result, it is necessary to design and analyze a polarization-insensitive THz-to-IR converter structure which is appropriate for incoherent sources, which is the main goal of this study.

Our starting point was to study the SRR structure given in Kuznetsov et al. [1,2] to understand the mechanisms behind absorption and IR emission. In our initial examination [5], ΔT is calculated for a similar SRR unit cell structure under constant coherent illumination by ignoring all loss mechanisms and assuming instantaneous absorption and IR emission. While this effort showed the successful conversion as proven by experiments, it was incomplete since it did not take into account loss mechanisms and response times to THz radiation and heat transfer. In this study, by using the same approach given in the work by Padilla et al. [3], we first calculate ΔT for the SRR structure by considering all loss mechanisms and the response time of the individual layers that make up the structure. Afterwards, since incoherent radiation is not typically linearly polarized, we also consider the performance of an MS unit cell design that can sense multiple linear polarizations. To eliminate the linear polarization dependence, a new cross-shaped unit cell design is examined and compared in performance to the SRR unit cell for THz-to-IR converter conversion [1,2]. Afterwards, both structures are compared for the case of incoherent illumination for the same illumination power as in the coherent case. It is found that the cross-shaped design performs with nearly similar performance as the SRR structure for linearly polarized illumination and that its performance increases at least by a factor of two for incoherent illumination. All calculated temperature differences are far greater than the limit of the minimum resolvable temperature difference for state-of-the-art IR microbolometer cameras. The degrading performance of the MS absorber layer for incoherent radiation for the same bandwidth of the source as in the coherent case results in a similar performance for ΔT in both cases. It is found that such dual-polarized or multi-polarization-sensitive MS absorber structures can be crucial for developing cost-effective converter structures which can be integrated with commercially available IR microbolometer cameras for concealed target detection. The overall passive device is only limited by its resolution due to the long wavelengths of THz radiation, as well as its response time, which, for the device structure analyzed here, is on the order of seconds for the converter structure based on the thin-film metallic MS layers described.

## 2. Metallic MS-Based THz-to-IR Converters

**Figure 2:**
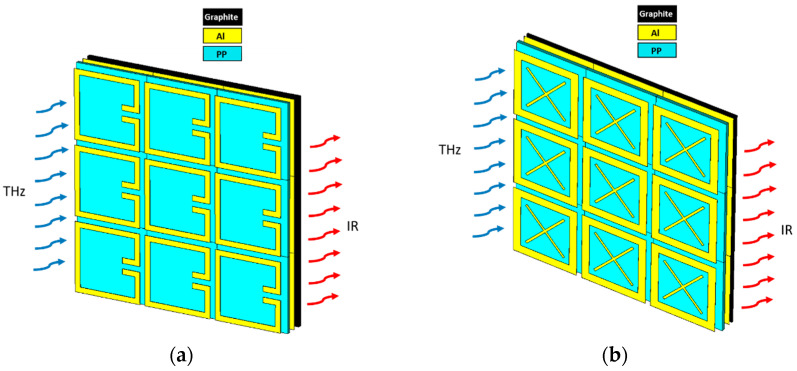
THz-to-IR converter array structures (thickness of Al, PP, and graphite are 0.4 µm, 20 µm, and 10 µm, respectively): (**a**) SRR; (**b**) cross-shaped structure.

**Figure 3:**
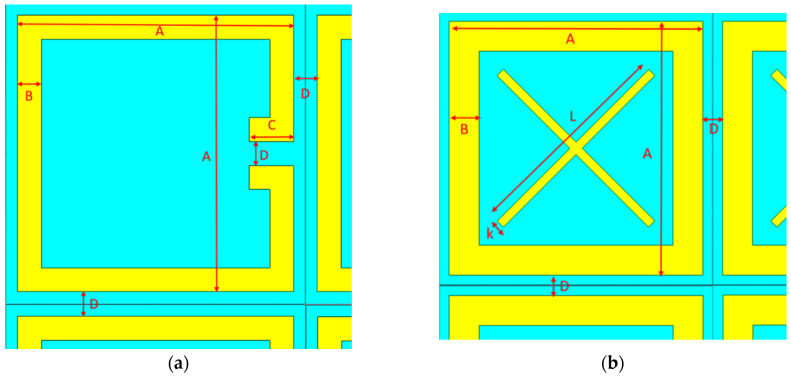
THz-to-IR converter dimensions: (**a**) SRR; (**b**) cross-shaped structure.

A comprehensive treatment of THz-to-IR converters is critical to understand the limits of these structures. It is important to point out that the studies here are carried out on metallic MS-based converter designs, which are relatively easier and cost effective to fabricate. The simple SRR structure studied by Kuznetsov et al. [1,2] fits this description and performs as a narrow-band polarization-sensitive THz-to-IR converter for terahertz (~0.3 THz) imaging. In this multilayer thin-film converter structure, a dielectric substrate, polypropylene film (PP) with thickness 20 µm, is used, and it is metallized from both sides with 0.4 µm thick Al to form highly conductive metallized ground and MS layers. The Al metasurface on the top works as a THz absorber, and a thin, highly emissive (ε = 0.93 [1,2]) graphitized layer on the bottom with a thickness 10 µm is used as an IR emitter. In this multilayered structure, the enhancement of IR emission from the emissive layer is provided by resonant absorption of THz waves causing converter heating, as shown in Figure 2. The dimensions of the SRR structure are given in Figure 3a and Table 1.

Alternatively, a new cross-shaped THz-to-IR converter structure which is on a metallic MS absorber layer is designed and its performance is analyzed. It has the same multilayered structure with the same materials and same thicknesses as the SRR structure. Differently, this structure has a cross (X)-shaped Al metasurface, as shown in Figure 3b, and the dimensions of the structure are also given in Table 1. Since this structure is symmetrical, it is dual-polarized and it is expected to have advantage in the case of an incoherent source when compared to the polarization-sensitive SRR-based structure. 

### 2.1. Analysis

Simulations are performed using commercially available software, Computer Simulation Technology Microwave Studio 2015 (CST-MWS), with the input material parameters given in Table 1 and Table 2 for the two structures [11]. 

**Figure 4:**
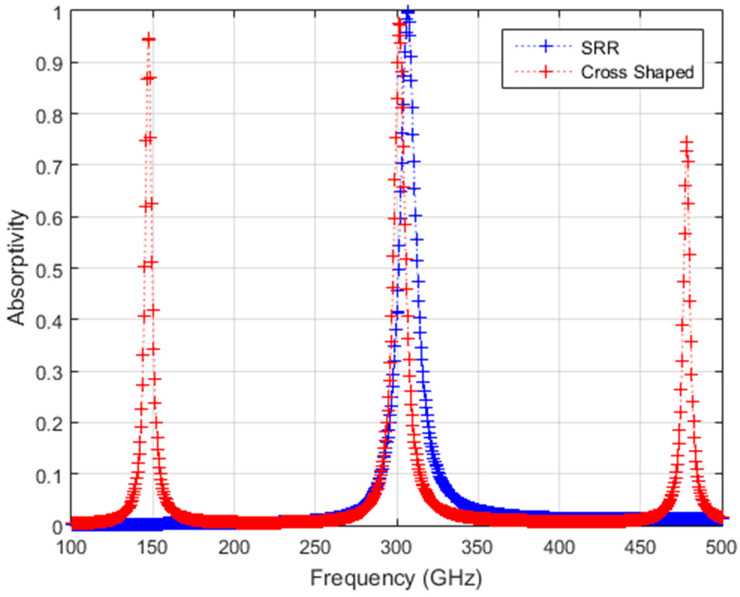
Calculated absorptivity (η_THz_ = 1 − |S_11_|^2^ − |S_21_|^2^) from CST simulations for SRR and cross-shaped structures.

SRR structure is polarization-sensitive, so a co-polar field illumination (E field is perpendicular to structure gaps) is used in the simulations. The simulations are performed using the frequency domain solver for the 0.1–500 GHz region, where atmospheric absorption is negligible, with the E-field in the x-direction with a high resolution of 0.5 GHz in order to effectively simulate the obtained temperature difference for THz sources with narrow emission bands. The transmission is negligible and the absorptivity (η_THz_) from the S_11_ and S_21_ values are calculated as η_THz_ =1 − |S_11_|^2^ − |S_21_|^2^. The simulated results are shown in Figure 4 for both structures.

Due to multiple gaps inside the cross-shaped structure with the outer ring, multiple resonances are expected compared to the SRR case, and this can be seen from Figure 4 where three resonance frequencies are clearly seen in the 0.1–500 GHz region. It is important to note that the bandwidth of the source used for subsequent calculations is not affected by the two absorption resonances to the left and right of the center resonance. To compare the cross-shaped structure with the SRR structure, we took only the resonance near 300 GHz in the following heat transfer analysis. From these simulations, an absorptivity, η_THz_, of 0.976 is found for the cross-shaped structure at 301.5 GHz, while an absorptivity of 0.997 is found for the SRR structure at 306.5 GHz, and this agrees with the MS simulation results for the SRR structure for the same geometrical parameters reported previously [1,2].

### 2.2. Temperature Difference Calculations

Bolometer-based IR camera detection is sensitive to the temperature difference, ΔT, due to changes in the IR radiative emission of the scene. It is necessary to study how the heat is transferred through these converters to calculate the temperature difference on the backside of the structure and to ensure that the THz power on the converter can produce sufficient temperature difference in the IR camera field. The minimum detectable thermal radiation is given by the camera’s Noise-Equivalent Temperature Difference (NETD) value. Initially, the front side metamaterial structure and backside are assumed to be at temperature T_0_ (ambient temperature), and when the front side absorbs incoming THz radiation power, the temperature increases from ambient temperature T_0_ to T_0_ + ∆T at the backside, causing a temperature difference, ΔT. The backside of the structure has high emissivity; therefore, it radiates some of its heat into the IR band of interest, and the IR bolometer camera can measure this temperature difference (Figure 2).

The heat balance equation is an accurate way to calculate ΔT since it considers all loss mechanisms that occur in the structure and also takes into account the thermal time constant, in other words, the response time of the converter.

#### 2.2.1. Heat Balance Equation

Here, the temperature difference obtained from heat balance equation is examined, which considers the thermal time constant and all loss mechanisms arising from conduction, convection, and radiation. The temperature difference in the detector is evaluated by solving the following heat balance equation [3,9]:

(1) $$C_{th}\frac{d(∆T)}{dt}+G_{th}∆T=\eta_{THz}∆\varphi$$

In the equation above, ∆φ is change in the incident flux, η_THz_ is the absorptivity of the front side metamaterial layer at THz frequencies (Figure 4), C_th_ is the thermal capacitance of the sensing element (J/K), and G_th_ is thermal conductance (W/K). If radiation power is not constant but is modulated at ω, then the incident flux becomes:

(2) $$\varphi \approx \varphi_{0}e^{i\omega t}$$

and then the temperature is also modulated at angular frequency ω,

(3) $$T\approx T_{0}e^{i\omega t}$$

If radiation the power and temperature equations above are inserted into Equation (1), it becomes:

(4) $$C_{th}i\omega ∆T_{0}e^{i\omega t}+G_{th}∆T_{0}e^{i\omega t}=\eta_{THz}{∆\varphi }_{0}e^{i\omega t}$$

(5) $$∆T\left(i\omega C_{th}+G_{th}\right)=\eta_{THz}∆\varphi$$

(6) $$∆T=\frac{\eta_{THz}∆\varphi }{G_{th}\left(1+i\omega \frac{C_{th}}{G_{th}}\right)}=\frac{\eta_{THz}∆\varphi }{G_{th}\left(1+i\omega \tau_{th}\right)}$$

where τ_th_ is thermal time constant needed to reach the steady state of the sensor defined as follows [9,14]:

(7) $$\tau_{th}=\frac{C_{th}}{G_{th}}$$

After some algebraic operations, ΔT can be written as [3,9,15]:

(8) $$∆T=\frac{\eta_{THz}{∆\varphi }_{0}}{G_{th}\sqrt{1+\omega^{2}{\tau_{th}}^{2}}}$$

In order to calculate and compare ΔT for the two THz-to-IR converter structures, thermal parameters (C_th_, G_th_, and τ_th_) also need to be calculated. In the following sections, the calculation methodology for these thermal parameters is explained in detail.

#### 2.2.2. Thermal Capacitance

Thermal capacitance, C_th_ (J/K), can be calculated by using the following equation [3,9]:

(9) $$C_{th}=\sum_{N}c_{th}\rho A_{0}d=\sum_{N}c_{th}\rho V$$

In this equation N is the number of different material layers of the sensing element, c_th_ is thermal heat capacity (J/kgK), ρ is density (kg/m^3^), V is the volume, and d is the thickness of each layer. A_0_ is the surface area of the sensing element, which is taken as 1 × 1 mm^2^ [16] for simplicity:

(10) $$C_{th}=A_{0}\left\{c_{Al}\rho_{Al}{(d}_{MS}+d_{GL})+c_{PP}\rho_{PP}d_{PP}+c_{Graphite}\rho_{Graphite}d_{EL}\right\}$$

By using Equation (10), thermal capacitance, C_th,_ is calculated as 23.13 µJ/K for both structures.

#### 2.2.3. Thermal Conductance

Total loss (Q_loss_) through conduction, convection, and radiation can be written as [3,9]:

(11) $$Q_{loss}=Q_{rad}+Q_{cond}+Q_{conv}$$

In the equation above, Q_cond_ is the power loss through conductive thermal transfer mainly in the supporting substrate, Q_conv_ is the power loss due to the free thermal convection to the air, which can be neglected (typically, the converter would operate inside a vacuum with the IR detector), and Q_rad_ is the power loss coming from the IR emission. All these loss mechanisms can be expressed in terms of the thermal conductance (G_th_) of total power loss, where Q_loss_ = G_th_ ∆T. Resultantly, G_th_ is evaluated from following equation:

(12) $$G_{th}=G_{rad}+G_{cond}$$

##### Radiative Loss

The radiative flux, Q_rad_ (W), is the radiometric quantity defined as the amount of optical power flowing into or out of a surface. The total radiative fluxes on both sides of the sensor can be derived as [9]:

(13) $$Q_{rad}=A_{0}\pi \int_{0}^{\infty }\varepsilon_{front}\left(\gamma \right)L_{\gamma }(\gamma ,T)d\gamma +A_{0}\pi \int_{0}^{\infty }\varepsilon_{\mathrm{bck}}\left(\gamma \right)L_{\gamma }(\gamma ,T)d\gamma$$

where ε_front_ and ε_bck_ are the emissivities of the layers on the front side and back side of the sensing element, and L_ν_ is the spectral radiance (W/m^2^ sr). For simplicity, it is assumed that the back side emissivity equals to the graphite emissivity ($\varepsilon_{bck}=0.93$) and the front side emissivity equals to zero ($\varepsilon_{front}=0$) [1,2,16]. Since T = T_0_ + ∆T, L_ν_ can be obtained from the Planck radiation law as follows [9,17]:

(14) $$Q_{rad}={\sigma A}_{0}\varepsilon_{\mathrm{bck}}\left(T^{4}-{T_{0}}^{4}\right)={\sigma A}_{0}\varepsilon_{\mathrm{bck}}\left[{\left(T_{0}+∆T\right)}^{4}-{T_{0}}^{4}\right]$$

In the equation above, σ is Stephan–Boltzmann constant (σ = 5.67 × 10^−8^ W/m^2^ K^4^) and T_0_ is the ambient temperature, which is taken as 300 K [16]. Using binomial expansion, the term in the parentheses can be expanded as follows [3,17]:

(15) $$Q_{rad}={\sigma A}_{0}\varepsilon_{\mathrm{bck}}\left[4{T_{0}}^{3}∆T\left(1+\frac{3}{2}\frac{∆T}{T_{0}}+{\left(\frac{∆T}{T_{0}}\right)}^{2}+\frac{1}{4}{\left(\frac{∆T}{T_{0}}\right)}^{3}\right)\right]$$

Since ΔT is very small compared to T_0_, the radiated power becomes [3]:

(16) $$Q_{rad}\approx {4\sigma \varepsilon_{\mathrm{bck}}A}_{0}{T_{0}}^{3}∆T$$

where Q_rad_ = G_rad_ ∆T and G_rad_ is calculated as 5.69 µW/K for both structures.

##### Conductive Loss

Loss due to thermal conductance is calculated by using the approach in study [16] as follows:

(17) $$Q_{cond}=G_{cond}∆T=4\kappa t∆T$$

In the equation above, κ is the heat conductivity of PP (κ=0.15 W/mK)—its high conductance is the reason for choosing it as the intermediary layer, and t is the thickness of the holder ($t=t_{pp}=20\;\mu m$) [16]. For simplicity, it is assumed that each holder only consists of PP film. By substituting all parameters, G_cond_ is calculated as 12 µW/K. Finally, by substituting the calculated radiative (G_rad_) and conductive (G_cond_) losses into the Equation (12), G_th_ is obtained as $G_{th}=17.69$ µW/K. 

In addition, the speed of a sensor is limited by the thermal time constant τ_th_, which is the response time of the converter, defined in Equation (7) [9]. This parameter is the time needed for the system to reach a steady state [14]. In other words, an equilibrium temperature is met, which is T = T_0_ + ∆T in our case [18]. For our structure, by using calculated C_th_ and G_th_ values, τ_th_ is found as 1.31 s. This value is higher than those for commercial state-of-the-art IR cameras, but it can be decreased by lowering the thickness of the structure and using materials having high thermal conductivity. 

#### 2.2.4. Efficiency

As stated in reference [16], THz-to-IR converter efficiency is defined as:

(18) $$eff=\frac{{\Delta Q}_{IR}}{Q_{THz}}=\frac{Q_{rad}}{Q_{cond}+Q_{rad}}$$

For SRR and our cross-shaped structure (G_rad_ = 5.69 µW/K and G_cond_ = 12 µW/K), the converter efficiencies are found as 32%. 

All of the calculated thermal parameters (C_th_, G_th_, τ_th_, and eff) for the SRR and cross-shaped structures are given in Table 3. These values are the same for both structures since the materials and thicknesses of the layers in the two structures are kept the same. 

#### 2.2.5. Temperature Difference Calculation for Coherent Source

In order to calculate ΔT from the equation obtained from heat balance equation, radiation source parameters are needed. The THz radiation source used in previous studies [1,2] is a backward oscillator (BWO) with tunable output wavelength and is reported to have a total of 5 mW emission power at a resonance frequency of 300 GHz. A typical bandwidth (∆f/f_peak_) of 15% is assumed and the peak frequency of the source is tuned to the resonance frequency of the MS layer. Then, the source spectral power density incident on the converter is assumed to have a Lorentzian-shaped function in the form of [19]:

(19) $$P\left(f\right)=\frac{P_{max}(f)}{1+{\left[\frac{\left(f-f_{peak}\right)}{FWHM}\right]}^{2}}$$

**Figure 5:**
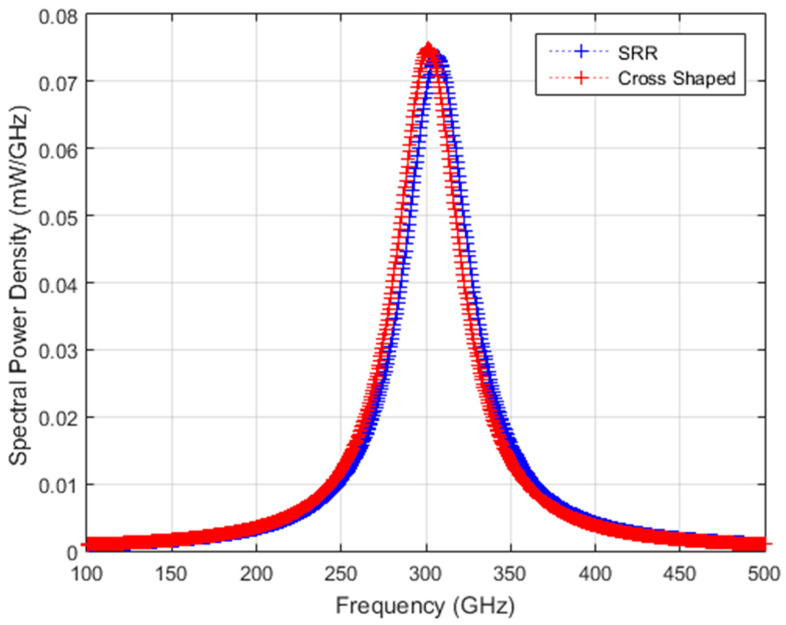
Spectral power density for a coherent Lorentzian-shaped source with a total power of 5 mW resonant with SRR and cross-shaped structures, respectively.

**Figure 6:**
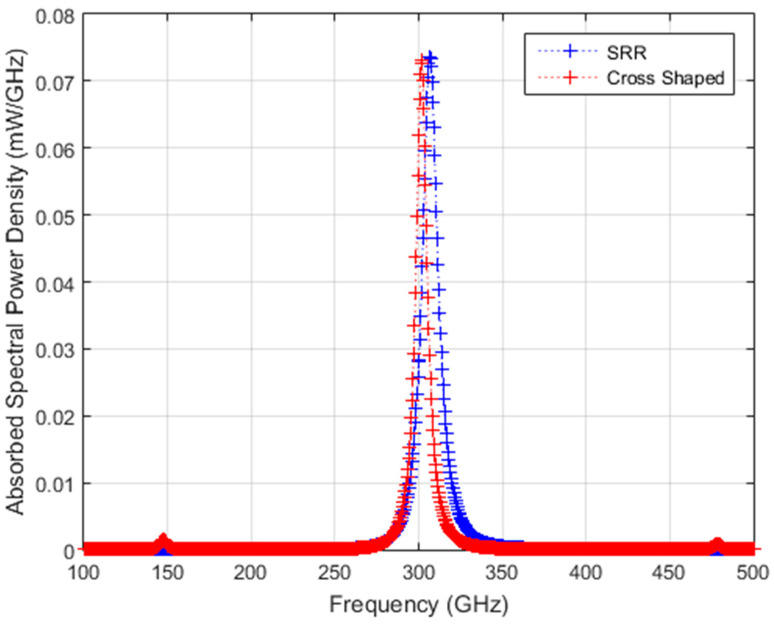
Absorbed spectral power density for a coherent Lorentzian-shaped source with a total power of 5 mW resonant with SRR and cross-shaped structures, respectively.

In the equations above, f_peak_ (GHz) is the resonance frequency of the converter structure; P_max_(f) is the maximum spectral power density observed for a source with a Lorentzian distribution that has a total power output of 5 mW at a center frequency of f_peak_; FWHM is the full width at half maximum (GHz), which is defined as ($FWHM=f_{peak}∆f/f$); and f is the frequency in GHz. For each converter structure, the source spectral power density is given in Figure 5. By multiplying absorptivity values, η_THz_, given in Figure 4 with the source power, an absorbed frequency-dependent spectral power density profile is obtained for the two structures for coherent source illumination, as shown in Figure 6. 

**Figure 7:**
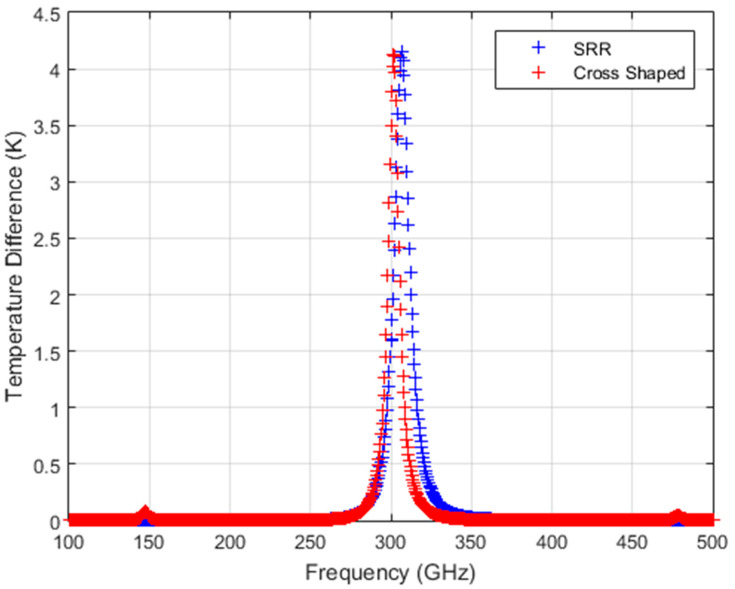
Calculated temperature difference (ΔT) from SRR and cross-shaped structures for a coherent Lorentzian-shaped source with a total power of 5 mW.

The ΔT curves of the two structures are calculated for the absorbed spectral power density (Figure 6) by substituting the calculated thermal parameters given in Table 3 into Equation (8). Frequency-dependent calculated ΔT curves are given in Figure 7 for both structures.

As can be seen in Figure 7, these results show that both converter structures give similar performance for linearly polarized illumination at 301.5 GHz and 306.5 GHz resonance frequencies, with 4.157 K and 4.130 K peak ΔT values for the SRR and cross-shaped structures with an area of 1 mm^2^, respectively. Since commercial state-of-the-art IR bolometer cameras can have NETD values on the order of 3 mK, the calculated ΔT values are well above the limit of the IR bolometer camera NETD values. 

#### 2.2.6. Temperature Difference Calculation for Incoherent Source

**Figure 8:**
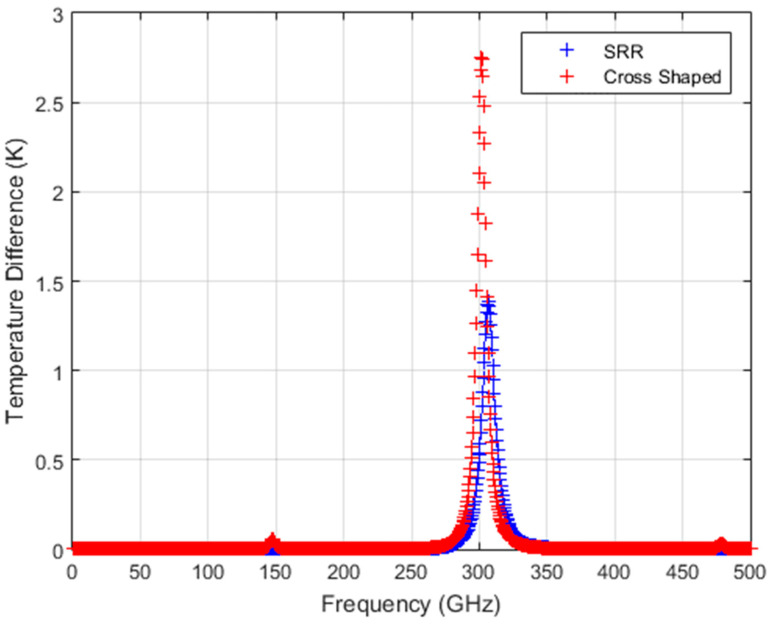
Estimated temperature difference (ΔT) from SRR and cross-shaped structures for an incoherent Lorentzian-shaped source with a total power of 5 mW.

The response of the two THz-to-IR converter structures is also calculated for the case of an incoherent source. This is similar to the radiation from natural sources which exists in our environment, as well as from lamps. For the incoherent source, the absorptivity of the two MS absorber structures will degrade when compared to the coherent illumination case. In the study by Droulias et al. [20], the performance of a metallic MS layer is analyzed for both the case of coherent and incoherent illumination. The simulated field is calculated for multiple linear polarization states while the unit cell orientation is kept stationary, and then the overall absorption of the structure can be estimated. For both the SRR and cross-shaped structures studied here, the effect of incoherent illumination resulted in an absorptivity that is about three times smaller and narrower than the original absorption for the coherent source illumination from the MS. For a dual-polarized sensitive MS absorber layer, the two polarization states will lead to a doubled sensing power, as has been previously shown in the study by [20], where the performance of a dual-polarized absorbing detector structure is compared to a single-polarized detector structure for incoherent source illumination. It is found that the dual-polarized device absorbs twice as much power compared to the single-polarized one. Applying the same analysis to the cross-shaped THz-to-IR converter structure for the detected power and assuming that due to the temperature increase the simulation material parameters are constant, which is fairly expected in this temperature range, the absorbed spectral power density and the calculated ΔT for a dual-polarized cross-shaped structure are therefore twice as great as those for the single-polarized SRR structure. Figure 8 shows the estimated temperature difference obtained from both structures for the Lorentzian-shaped incoherent source with a total power of 5 mW. The results for the incoherent source show that in the dual-polarized cross-shaped structure, the calculated ΔT increases to 2.753 K, which is two times higher than that for the SRR structure. Due to the dual-polarized nature, the resultant increase in ΔT enables better detection with IR cameras of incoherent THz sources. The comparisons of the calculated ΔT values for the two THz-to-IR converter structures for incoherent and coherent source illuminations are presented in Table 4.

## 3. Conclusions

THz emission from thermal sources is a field which continues to attract interest due to the many new applications that arise from a remote sensing perspective. The development of polarization-insensitive THz-to-IR converters can be extremely beneficial for detection of naturally occurring THz radiation and low-IR-signature target detection. Here, a new cross-shaped MS unit cell-based absorber is shown to be effective in the detection of incoherent THz radiation. The performance of this structure is compared with that of the SRR unit cell-based MS absorber structure that previously has been shown to be effective in the detection of linearly polarized coherent THz radiation [1,2]. A model is developed to calculate the temperature difference due to coherent and incoherent source radiation incident on the converter considering all loss mechanisms and the response time of the structures. The results show that under linearly polarized coherent illumination, both structures show similar temperature difference values of around 4 K at resonance frequencies around 300 GHz for a Lorentzian-shaped coherent source with a total power of 5 mW. For an unpolarized incoherent source under similar illumination considerations, the MS absorber layer’s performance decreases by about a factor of three; however, due to the nature of the cross-shaped structure design being sensitive to dual-polarizations, it still exhibits a temperature difference of 2.75 K, which is at least two times greater than that of the SRR structure for an illuminated area of 1 mm^2^. Depending on the intensity of the incoherent source and size of the converter structure, these results suggest that the resulting temperature differences can be well detected with current state-of-the-art IR cameras with minimum resolvable temperature difference sensitivity on the order of a few mKs. The use of an MS absorber layer with a broader absorptive resonance can be more useful for incoherent detection of THz radiation but would be less beneficial for detection of a narrowband coherent source. While the response time of the structure, which was found to be on the order of 1 s, can be improved using lighter materials with higher thermal conductivity, the sensitivity of this unique THz-to-IR converter makes it especially beneficial for low-IR-signature target detection since atmospheric transmission losses in the sub-500 GHz range are negligible.

## Figures and Tables

**Table 1:** Dimensions of THz-to-IR converter structures (t_pp_: thickness of PP; t_graphite_: thickness of graphite; t_Al_: thickness of Al).

Structure	A (µm)	B (µm)	C (µm)	D (µm)	L (µm)	k (µm)	t_pp_ (µm)	t_graphite_ (µm)	t_Al_ (µm)
SRR	102	12	27.5	8	-	-	20	10	0.4
Cross-shaped	408	48	-	32	344	14	20	10	0.4

**Table 2:** Input material parameters taken in CST simulations and temperature difference calculations [11,12,13].

	PP	Graphite	Al
ϵ_r_ (relative permittivity)	2.2	-	-
µ_r_ (relative permeability)	1	-	1
tanδ (loss parameter)	0.0005	-	-
Thermal conductivity (W/K/m)	1.58	-	237
Electrical conductivity (S/m)	-	-	3.56 × 10^7^
C_th_ (heat capacity (kJ/K/kg))	0.27	0.72	0.9
Thermal expansion coefficient (1/K)	-	-	23 × 10^−6^
ρ (density (g/cm^3^))	0.9	2.267	2.710

**Table 3:** Calculated thermal parameters for SRR and cross-shaped structures ($\varepsilon_{bck}=0.93$, $\;\varepsilon_{front}=0$, T_0_ = 300 K, and A_0_ = 1 × 1 mm^2^).

C_th_ (µJ/K)	G_rad_ (µW/K)	G_cond_ (µW/K)	G_th_ (µW/K)	τ_th_ (s)	eff (%)
23.13	5.69	12	17.69	1.31	32

**Table 4:** Comparison of the maximum absorptivity (η_THz-max_), maximum absorbed spectral power density (P_max_), and temperature difference (ΔT) values obtained from SRR and our cross-shaped structures for coherent and incoherent Lorentzian-shaped sources with a total power of 5 mW.

Structure	Source	η_THz-max_ (f)	f_peak_ (GHz)	P_max_(f) (mW/GHz)	ΔT (K)
SRR	Coherent	0.997	306.5	0.074	4.157
Cross-Shaped		0.976	301.5	0.073	4.130
SRR	Incoherent	0.332	306.5	0.024	1.386
Cross-Shaped		0.325	301.5	0.024	2.753

## Data Availability

Data are contained within the article.
